# The Relative Effectiveness of Bilateral and Unilateral Electrode Placement in Electroconvulsive Therapy (ECT) in Patients With Major Depressive Disorder: A Retrospective Cohort Study

**DOI:** 10.7759/cureus.42938

**Published:** 2023-08-04

**Authors:** Tatjana Balint, R. Nazim Khan, Geoff Hooke

**Affiliations:** 1 Psychiatry, Oceania University of Medicine, Apia, WSM; 2 Psychiatry, Perth Clinic, Perth, AUS; 3 Mathematics and Statistics, School of Physics, University of Western Australia, Perth, AUS; 4 Information Technology and Research, Perth Clinic, Perth, AUS; 5 Psychological Science, University of Western Australia, Perth, AUS

**Keywords:** depression anxiety and stress scale (dass-21), dass-21 scale, major depression disorder, dass-depression scoring scale, patient demographics, unilateral electrode placement, bilateral electrode placement, electroconvulsive therapy (ect)

## Abstract

Background

This study is focused on the comparative efficacy of bilateral and unilateral electroconvulsive therapy (ECT) on depressive symptoms in patients at the Perth Clinic for the period from 2016 to 2021.

Methods

This was a retrospective cohort study of 485 patients who received ECT treatment. The expected improvements in depressive symptoms were evaluated by the Depression Anxiety Stress Scale (DASS) assessment tool filled out by the patients on admission and discharge from the hospital. Only the depression score of the DASS scale was utilised for this research.

Results

The results suggested that both electrode placements resulted in a significant improvement in depressive symptoms. The positive response rates for the bilateral and unilateral electrode placements were 78.3% and 71.6%, respectively. There was no difference between males and females in the average DASS score at discharge for bilateral and unilateral electrode placements.

Conclusions

This study confirmed that the results obtained at the Perth Clinic are similar to the existing international research results on the same topic. Bifrontal and unilateral ECT electrode placements are equally efficacious in improving depressive symptoms in patients suffering from major depressive disorder (MDD).

## Introduction

Attempts to treat mental health disorders by inducing seizures were recorded as early as the 16th century, when Swiss alchemist Paracelsus gave patients camphor oil by mouth to induce convulsions to "cure lunacy". This approach became well accepted during the 18th and 19th centuries, when several cases of effective treatments were documented. Based on the previous studies on seizure induction in dogs by Swiss researchers, two Italian scientists, Ugo Cerletti and Luigi Bini, developed the idea of applying electrical current to the scalps of human patients in order to provoke generalised epileptic-type seizures [1].

Cerletti and Bini gave the first demonstration of electroconvulsive therapy (ECT) in Rome in 1938 on a man who was found to be delusional at a train station. Such treatment was considered a great success, and the patient was discharged as recovered after 11 sessions. After Europe, ECT spread fast in the USA in the 1940s. While early application of this method was confined to the treatment of schizophrenia, from the late 1940s on, ECT was applied for the treatment of major depressive disorder (MDD) as well. With the development of new pharmaceutical products applied to mental health, the use of ECT somewhat declined in the 1970s and 1980s, partly due to the stigmatisation and perception of this method as a "coercive" and "last resort" treatment [1].

Contemporary ECT remains conceptually similar to Cerletti and Bini’s initial work. The advances in brain imaging, post-mortem studies, and applied advances in anaesthesia, pharmacotherapy, the application of pulse waves, and focused hemi-scalp stimulation rather than whole-brain involvement all contributed to the more effective and safer application of ECT in the management of not only psychoses but mood disorders as well [2].

Electroconvulsive therapy is currently considered to have unparalleled antidepressant efficacy despite its possible cognitive side effects in patients suffering from MDD. More recent remission rates are estimated to be in the range of 50%-70% [3].

It is now widely recognised that ECT-induced tonic-clonic seizures are necessary for both the beneficial and adverse effects of treating depression. Other parameters such as seizure duration, electric stimuli seizure threshold, ECT practice factors, concomitant use of medications, and other factors may also affect patient outcomes. In recent years, more extensive research has been conducted about the role of neurotransmitters, new genetic tools have been utilised, and structural and functional neuroimaging techniques have been developed. Despite the research efforts addressing the mechanisms underlying the effect of ECT treatment, the general mechanisms of action still remain poorly understood [4].

Petinatti and Nilsen [5] conducted a quantitative procedure meta-analysis of the literature assessing the efficacy of bilateral versus non-dominant unilateral ECT electrode placements. The research also involved the evaluation of as many as eleven variables, which span research methodology, technique, patient demographics (age, sex), and operational dimensions. The results supported the conventional view that no difference existed between the two electrode placements in terms of patient outcomes [5].

A few differences have been proven between ECT placements, and the ones that were reported could be accounted for by the differences in electric stimulus dosage. Ottoson [6] published a survey of 24 comparisons of the anti-depressive efficacy of bilateral versus unilateral electrode placements. The results showed a preponderance of studies in favour of bilateral ECT. However, the inferior outcomes of the unilateral ECT could also be explained by submaximal seizure activity due to the short inter-electrode distance, as well as the effect of medications such as benzodiazepines with ECT.

Tandon et al. [7] compared the efficacy of unilateral versus bilateral ECT in melancholia in 46 non-randomly assigned, medication-free patients with endogenous depression. By utilising blind assessments on the Hamilton Rating Scale for Depression, they showed a 57% improvement in the bilateral electrode placement group compared to a 19% improvement in the unilateral group [7]. The authors found that, while unilateral ECT may induce fewer side effects, bilateral treatment was more efficacious in the short term.

Weiner and Coffey found that conflicting results about the efficacy of unilateral versus bilateral electrode placements in ECT continue to be reported [8]. The authors concluded that the therapeutic advantages of bilateral electrode utilisation in ECT may be related to the use of non-optimum unilateral ECT techniques. Their opinion was that improved electrode location, electrode-scalp contact interface changes, stimulus dosing adjustments, and better seizure monitoring may allow unilateral ECT to be utilised with maximal therapeutic potency.

By using a randomized controlled study with computer modelling, Martin et al. [9] investigated the effects of both electrode placement and pulse width on cognitive side effects with unilateral ECT. By investigating nine participants, it was found that frontoparietal electrode placement was not superior to temporoparietal for memory outcomes. Their preliminary findings suggested that higher electric fields may be associated with greater cognitive ECT side effects.

Spiric et al. investigated the effect of demographics on ECT outcomes, and they found that significant improvement after the use of electroconvulsive therapy was associated with sex but not with age [10]. Age was noted to have a significant impact on the efficacy of ECT in a study by Birkenhager et al. [11]. In the study by Bolu et al., ECT as a treatment for MDD was equally effective in women and men [12].

In this study, we investigated the effect of unilateral versus bilateral electrode placement on depressive symptoms in patients with MDD treated at the Perth Clinic during a five-year period between 2016 and 2021.

## Materials and methods

This was a retrospective cohort study of patients aged between 16 and 83. A total of 485 patients included in the study received ECT therapy between 2016 and 2021 at Perth Clinic, which is a private psychiatric hospital in the Perth Metropolitan area of the Western Australian capital city of Perth. The data were obtained from the existing patient database at the Perth Clinic for the above period.

The ethical approval for this study was granted by the Institutional Review Board of the Oceania University of Medicine, Apia, Samoa (approval number 22-0412TB). The patients for this study were selected based on the criteria of having a diagnosis of medication-resistant major depressive disorder, defined as having an inadequate response to at least two trials of optimal antidepressant pharmacotherapy. All the patients receiving ECT had signed informed consent prior to treatment.

Initially, 640 patients with major depressive disorders were included in the study. The data of 130 patients was excluded due to a lack of DASS scores either on admission or at discharge. Twenty-five patients who had changes in electrode placement during ECT were also excluded. Changes were made in regards to their seizure threshold, the applied electric pulse width, the pulse frequency, and seizure duration recorded on electroencephalogram (EEG) tracing.

The patients had all received ECT treatment using a Thymatron® System IV (Somatics, LLC, Lake Bluff, IL, USA). The treatments were applied three times a week. The electrode placements for ECT administrations were either bilateral (bifrontal and bitemporal) or unilateral (right-unilateral). The treatments were administered in line with local clinical guidelines and were rated as effective.

**Figure 1:**
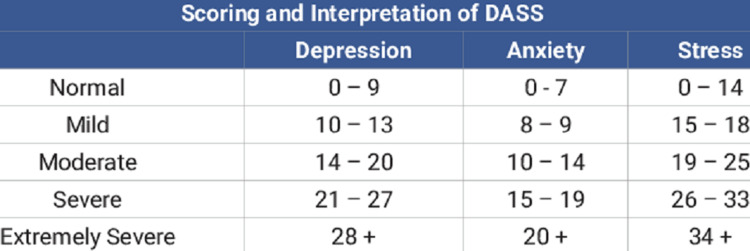
The severity rating index of the Depression Anxiety and Stress Scale (DASS)

Any improvements in depressive symptoms in patients were evaluated via the patient self-completed Depression Anxiety Stress Scale (DASS), measuring only the depression scores (D) pre- and post-ECT application (Figure 1).

The DASS scale is a self-reported mood assessment tool that measures negative emotional states of ‘D’-depression, ‘A’-anxiety, and ‘S’-stress. Each of the three DASS scales contains seven items divided into subscales with similar content. The depression scale assesses hopelessness, dysphoria, lack of interest, lack of involvement, anhedonia, inertia, and devaluation of life. The score for depression is calculated by adding up the scores for the relevant items.

Data analysis, using graphs and numerical summaries, was conducted. Two statistical models were fitted to the data. First, the difference in the DASS score was computed as:

DASS-Diff = (DASS score post-ECT) - (DASS score pre-ECT)

where DASS-pre and DASS-post are the DASS scores before and after the respective treatments. The focus of the analysis was the effect of ECT placement on the difference in DASS scores.

1. A linear statistical model [13] was fitted to DASS at discharge against the ECT placement and demographic variables (age and sex). This model included the three ECT placements: bifrontal, bitemporal, and unilateral.

2. The same linear statistical model [13] was fitted to DASS-Diff against ECT placement and demographic variables. The DASS-pre was included as a moderator in the model, as it is expected that the difference in DASS will depend on its initial value. This model used bilateral and unilateral ECT placements; that is, the bifrontal and bitemporal electrode placements were merged.

All statistical analysis was conducted in the R statistical environment (R Foundation for Statistical Computing, Vienna, Austria) [13]. Statistical significance was taken at 5% (p = 0.05).

## Results

Demographics

**Figure 2:**
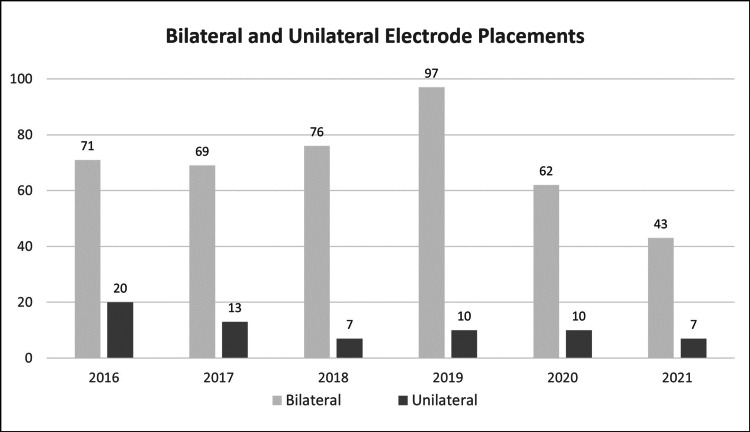
The number of patients treated with bilateral and unilateral electrode placements (2016–2021)

**Figure 3:**
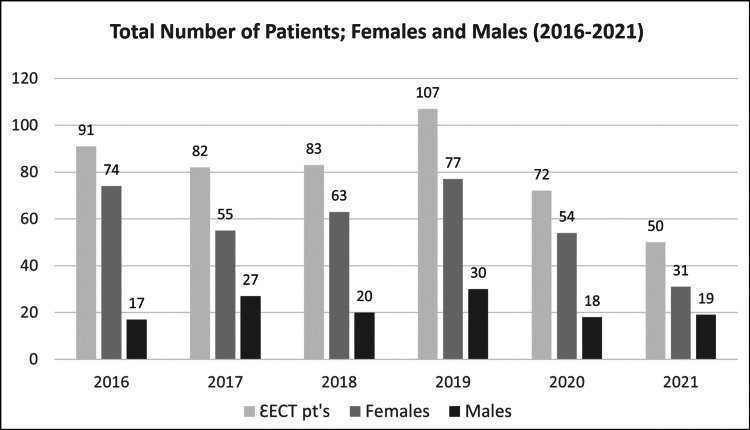
Total number of patients and their division by sex (2016–2021)

A total of 485 patients with medication-resistant depression received ECT treatment at the Perth Clinic between 2016 and 2021; 419 male and female patients were treated with bilateral ECT, while the remaining 66 patients received ECT via unilateral electrode placement (Figures 2-3, Table 1).

**Table 1:** Number of patients with depression DASS scores on admission and discharge basis bilateral and unilateral electrode placements, and main percentages (%) DASS: Depression Anxiety Stress Scale

	Bilateral			Unilateral	
Depression DASS score	Number of Patients (Admission DASS), %	Number of Patients (Discharge DASS), %	Depression DASS Score	Number of Patients (Admission DASS), %	Number of Patients (Discharge DASS), %
Severe Depression	119 (24.8%)	9	Severe Depression	11 (16.6%)	4
Moderate Depression	207 (49.4%)	80	Moderate Depression	42 (63.3%)	15
Mild Depression	47	77 (18.3%)	Mild Depression	10	14 (21.2%)
Normal Mood	46	253 (60.3%)	Normal Mood	3	33 (50%)
Normal Mood and/or Mild Depression	93	330 (78.3%)	Normal Mood and/or Mild Depression	13	66 (71.2%)

The variables in the dataset and some summaries are listed in Table 2.

**Table 2:** A summary of DASS scores on admission and discharge, categorised by sex and ECT placement, and age and ECT placement; the number of cases is given in brackets. DASS: Depression Anxiety Stress Scale; ECT: electroconvulsive therapy

		DASS Score: Admission				DASS Score: Discharge			
		Bifrontal (308)	Bitemporal (111)	Unilateral (66)	Overall	Bifrontal	Bitemporal	Unilateral	Overall
Minimum	Male (132)	1	10	9	1	0	2	0	0
	Female (353)	2	2	1	1	0	0	0	0
	Overall	1	2	1	1	0	0	0	0
Mean	Male	15.7	17.3	16.6	16.1	8.2	10.7	8.7	8.6
	Female	17.0	17.0	17.1	17.0	8.8	6.6	10.5	8.5
	Overall	16.6	17.1	17.0	16.8	8.6	7.4	10.0	8.5
Median	Male	17.5	17.5	17.5	17.0	7.0	10.5	7.0	7.5
	Female	19.0	19.0	19.0	19.0	8.0	5.0	11.0	8.0
	Overall	18.5	19.0	18.5	19.0	8.0	6.0	9.5	8.0
Maximum	Male	21	21	21	21	21	20	19	21
	Female	21	21	21	21	21	21	21	21
	Overall	21	21	21	21	21	21	21	21
		Age			
Age	Minimum	18	16	18	16
	Mean	43.3	47.0	45.9	44.5
	Median	44.0	48.0	47.5	45.0
	Maximum	83.0	78.0	68.0	83.0

Numerical and graphical summaries of the data were obtained. We fitted a linear statistical model (by Julian J. Faraway) to the DASS score at discharge against the other variables in the data [13].

Of the 419 patients treated with bilateral ECT, 326 (74.2%) had moderate and/or severe depression scores on the DASS scale at admission. On the DASS scale on discharge, 253 (60.3%) of those patients scored/had a normal mood, and 330 (78.3%) of them scored normal and/or mild depression (Table 1).

Of the 66 patients treated with ECT using unilateral electrode placements, 53 (79.9%) had scores of severe and/or moderate depression on the DASS scale at admission. On the DASS scale on discharge, 33 patients (50%) had scores of normal mood, or 47 (71.2%) of them had scores of normal mood and/or mild depression (Table 1).

Statistical results

Numerical and graphical summaries of the data were obtained. We fitted a linear statistical model to the DASS score at discharge against the other variables in the data [13]. The admission DASS score was used as a moderator in the model, since the change in the DASS score at discharge may depend on the initial value. A table of the significant variables is shown in Table 2.

The analysis showed the following:

1. The number of days in hospital (closely related to the number of ECT treatments) had no significant effect on the DASS score at discharge (statistical analysis of the number of days in hospital: minimum = 3, mean = 28.15, median =26, maximum = 83).

2. Older patients had a lower discharge score of 0.06 per year. A patient who was 10 years older had a lower mean score of 10 x 0.6 =0.6. Given the age range of 67, the mean difference between the youngest and oldest patients was 67 x 0.06 = 4.2.

3. An increase in the DASS admission score of 1 resulted in an average increase in the discharge DASS score by 0.34. Since the range of the DASS admission score is 20 (Table 2), this indicates a mean difference of 20 x 0.34 =6.8 in the discharge score between the lowest and highest admission scores.

**Figure 4:**
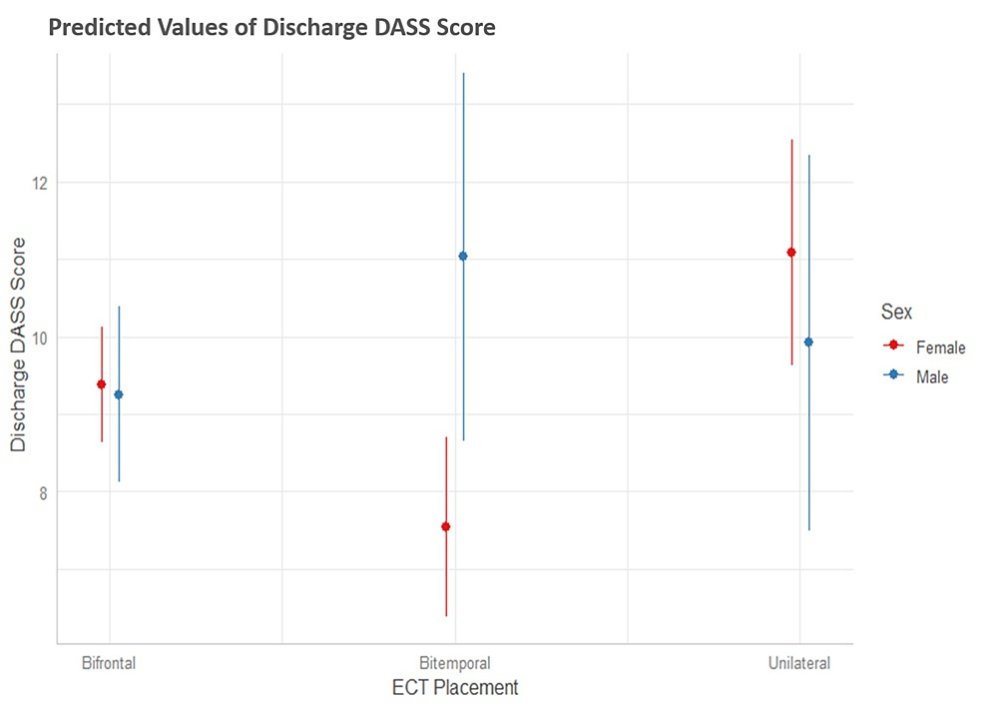
A model-based interaction plot of DASS score at discharge with the sex of the patients and electrode placements DASS: Depression Anxiety Stress Scale; ECT: electroconvulsive therapy

The interesting result was that there was an interaction between sex and ECT placement, as presented in the graph in Figure 4.

Figure 4 shows that there was no difference between males and females in the average DASS score at discharge for bilateral and unilateral placements, but males with bitemporal placement had a higher mean score of 3.6 (Table 3) compared to females with bitemporal placement.

**Table 3:** The result of the linear statistical model for DASS at discharge DASS: Depression Anxiety Stress Scale; ECT: electroconvulsive therapy

Variable	Estimate	Standard Error	p-value
Sex	-0.14	0.6650	0.8302
Age	-0.06	0.0170	0.0002
DASS Admission	0.34	0.0516	1.4 x 10^-10^
ECT Placement			
Bilateral	-1.85	0.6700	0.0140
Unilateral	1.70	0.8632	0.0495
ECT Placements by Sex of the Patients			
Male: Bilateral	3.64	1.4762	0.0140
Male: Unilateral	-1.03	0.8632	0.5126

We were interested in the difference in discharge DASS score between bilateral (that is, bifrontal or bitemporal combined) and unilateral placements. The summary statistics for the number of patients and discharge DASS score by ECT placements are given in Table 4.

**Table 4:** Summary statistics for DASS-Diff by ECT placement DASS: Depression Anxiety Stress Scale; ECT: electroconvulsive therapy

	Bilateral	Unilateral
Number of Patients	419	66
Mean	8.3	9.0
Standard Deviation	5.73	5.71

The mean discharge DASS score for unilateral placement was higher compared with bilateral placement, with no difference in standard deviations.

**Figure 5:**
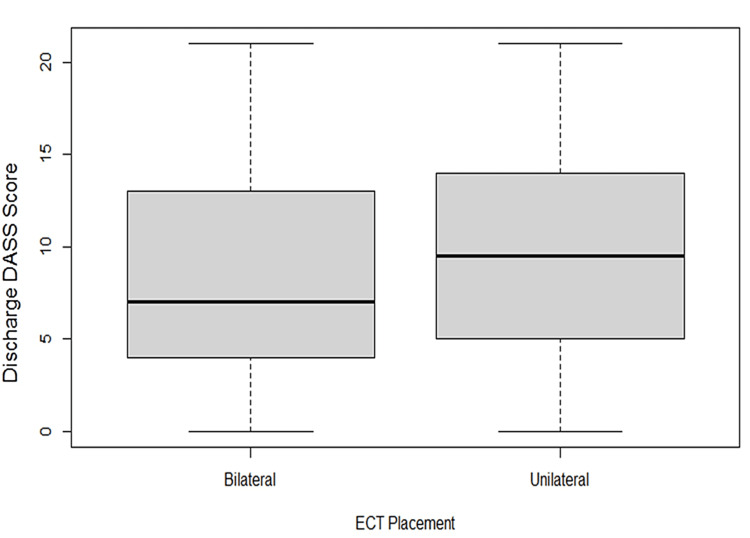
A boxplot of the DASS discharge score by ECT placement, which is categorised as bilateral or unilateral DASS: Depression Anxiety Stress Scale; ECT: electroconvulsive therapy

An analysis of variance (ANOVA) (equivalent to two independent sample t-tests) was fitted to the DASS scores with ECT placements categorised as bilateral and unilateral. The model shows a difference in median scores (Figure 5) by ECT placement (p-value = 0.0128); that is, the median DASS discharge score is higher for unilateral electrode placement by 1.7 compared with bilateral placement.

## Discussion

Our main interest in this study was to evaluate the comparative effectiveness of two different types of electrode placements in managing depression in patients at the Perth Clinic.

The difference in the effectiveness of two different delivery methods of ECT in the Perth Clinic was statistically significant (mean difference in discharge DASS score of 1.7), but not clinically significant. The response rates for bilateral placement were 60% and/or 78%, and for unilateral placement, 50% or 71% (Table 1), percentages of patients with normal mood and/or normal and mild depression on discharge in two different groups. Similar results were reported in the study by Bjolset et al., where formula-based bilateral and right unilateral ECT were equally efficacious in a clinical sample of elderly patients with major depression [14].

This study has some weaknesses. Firstly, the number of patients in the unilateral group was only 66, compared with 419 in the bilateral group. Data exploration shows that the range of DASS admission scores for these two groups is different. In particular, patients with bilateral electrode placement had lower admission DASS scores than patients with unilateral electrode placements.

In the systematic review work on the cognitive effects of ECT by Kumar et al., it was indicated that unilateral electroconvulsive therapy (RUL) was deemed less effective, while in our study, right unilateral therapy was showing almost equal efficacy as bilateral therapy [15].

The analyses in some other studies suggest that unilateral electrode placement tends to increase the average number of treatments required for recovery compared to bilateral electrode placement. This could be the reason why the bilateral method of delivering ECT to patients in the Perth Clinic was the preferred method. In our study, however, DASS scores on discharge were not impacted by length of stay (or average number of treatments).

The lower number of patients with unilateral placements in our study could be due to exclusion at the start; they were switched from unilateral to bilateral placements because of insufficient progress throughout the ECT course (that was one of our exclusion criteria). A similar tendency to switch to another placement was reported by D’Cunha et al., where a quarter of the studied subjects had to switch to bilateral placement [16].

Our results will contribute to further improving the current evidence-based therapeutic guidelines used at the Perth Clinic.

Limitations

These results could not be generalised for the wider population as it is an observational study. A designed study is required, with patients randomly assigned to one of the placements and matched for demography and diagnosis, to confirm these results.

## Conclusions

This study indicates that both methods, bilateral and unilateral electrode placement in electroconvulsive therapy (ECT), are equally efficacious in treating patients with medication-resistant major depressive disorder. However, the results cannot be generalised to the wider population due to the weaknesses discussed above. A better-designed study would be required to confirm this result.
